# TGF beta and IL13 in schistosomiasis mansoni associated pulmonary arterial hypertension; a descriptive study with comparative groups

**DOI:** 10.1186/1471-2334-14-282

**Published:** 2014-05-21

**Authors:** Rita de Cassia dos Santos Ferreira, Silvia Maria Lucena Montenegro, Ana Lucia Coutinho Domingues, Angela Pontes Bandeira, Carlos Antonio da Mota Silveira, Luiz Arthur Calheiros Leite, Clara de Almeida Pereira, Izolda Moura Fernandes, Alessandra Brainer Mertens, Milena Oliveira Almeida

**Affiliations:** 1 Department of Tropical Medicine, Health Sciences Center, Universidade Federal de Pernambuco, Recife, Brazil; 2 Department of Immunology, Centro de Pesquisas Aggeu Magalhães - Fundação Oswaldo Cruz, Recife, Brazil; 3 Departament of Clinical Medicine, Health Sciences Center, Universidade Federal de Pernambuco, Recife, Brazil; 4 Reference Center of Pulmonary Hypertension, Pronto Socorro Cardiológico de Pernambuco, Universidade de Pernambuco, Recife, Brazil; 5 Department of Biochemistry, Health Sciences Center, Universidade Federal de Pernambuco, Recife, Brazil; 6 Biological Sciences Center, Universidade Federal de Pernambuco, Recife, Brazil

**Keywords:** Schistosomiasis, Pulmonary hypertension, Transforming growth factor-beta, Interleukin 13

## Abstract

**Background:**

It is suggested that interleukin (IL)-13 and transforming growth factor (TGF)-beta play a role in the pulmonary vascular changes found in animal models of schistosomiasis. The aim of this study was to assess and compare the serum levels of total TGF-beta and IL-13 of patients with schistosomiasis with pulmonary arterial hypertension (PAH) and patients with schistosomiasis without PAH.

**Methods:**

34 patients from the schistosomiasis outpatient clinic of the Hospital das Clinicas, Recife, Pernambuco, Brazil, without PAH assessed by echocardiography and 34 patients from the Reference Centre of Pulmonary Hypertension of Pronto Socorro Cardiológico de Pernambuco, Recife, Brazil with PAH, confirmed by right heart catheterization, were enrolled on the study. Both groups presented with schistosomal periportal fibrosis after abdominal ultrasound. Serum levels of TGF-beta1 and IL-13 were determined by ELISA. Student t test to independent samples, Mann-Whitney test to nonparametric variables, Pearson correlation test for correlation analyses and Fisher Chi-squared test to compare categorical analyses were used.

**Results:**

The median value of TGF-beta1 was significantly higher in patients with PAH (22496.9 pg/ml, interquartile range [IR] 15936.7 – 32087.8) than in patients without PAH (13629.9 pg/ml, IR: 10192.2- 22193.8) (p = 0.006). There was no difference in the median value of IL-13 in the group with Sch-PAH compared to patients without Sch-PAH (p > 0.05).

**Conclusion:**

Our results suggest that TGF-beta possibly plays a role in the pathogenesis of schistosomiasis-associated PAH.

## Background

Pulmonary arterial hypertension (PAH) is defined hemodynamically as an increase in mean pulmonary arterial pressure (PAP) ≥ 25 mmHg at rest and pulmonary artery occlusion pressure ≤ 15 mmHg, characterized by the remodelling of the pulmonary vasculature that causes progressive dyspnea and can lead to right-sided heart failure and premature death [1]. It may be caused by several conditions including idiopathic PAH, connective tissue disease, HIV infection, schistosomiasis and others [2]. Around 240 million people are infected with some species of *Schistosoma* throughout the world, mainly *S. japonicum, S. haematobium* and *S. mansoni*, the latter being responsible for almost all cases of schistosomiasis-associated pulmonary arterial hypertension (Sch-PAH) [3-5]. Sch-PAH represents 30.8% of all causes of PAH in endemic areas of *S. mansoni*, and is considered the leading cause of PAH in these areas [6]. Hospital-based studies in Brazil, where *S. mansoni* is endemic, have reported pulmonary hypertension in approximately 10% of patients with schistosomal periportal fibrosis, using echocardiographic assessment [7,8], but only 4.7% of patients with the hepatosplenic form of the disease presented PAH confirmed by right heart catheterization (RHC) [8].

The main lesion caused by *S. mansoni* is due to an intense immune reaction against the parasite eggs trapped in small branches of the intrahepatic portal vein, resulting in chronic granulomas that stimulate the development of fibrosis in the portal spaces [5,9]. Granuloma formation is a predominantly T helper (Th) cell-dependent process, with an early Th1 proinflammatory response (IL-1, IL-12, interferon gamma, transforming growth factor beta [TGF – beta] and tumour necrosis factor-alfa), in which a switch occurs following egg production to a predominant Th2 immune response (IL-4, IL-5, IL-10 and IL-13) that plays a central role in the modulation of granuloma immune response [10-14].

Depending on the intensity of the fibrotic reaction around the portal veins, almost 5-10% of chronically infected individuals will suffer blockage of the hepatic blood flow and secondary portal hypertension [15]. The downstream effect is when the naturally occurring portal-systemic collateral vessels open to decompress the portal system [16,17], which allows embolization of eggs to the lungs. A secondary granulomatous reaction is triggered in the pulmonary vascular bed and gives rise to obliterative arteritis and vascular remodeling, which in some individuals results in PAH [18]. This is the main mechanism which is generally believed to be related to the development of Sch-PAH. However, a recent study failed to demonstrate a significant amount of parasite-derived antigens within the lungs of individuals who had died of Sch-PAH [19], but the histologic and vascular changes encountered were similar to those described in idiopathic PAH [20]. This suggests that embolization of eggs to the lungs is not the sole mechanism responsible for establishing PAH [19].

It is possible that genetic determinants are involved in the development of the disease. Around 80% of patients with familial PAH and 25% with idiopathic PAH present mutations in the gene encoding the bone morphogenetic protein receptor type II (BMPR-II), a member of the TGF-beta superfamily [21]. This leads to a change in the signaling of the TGF-beta superfamily. There is strong evidence to suggest that the TGF-beta system, by stimulating the proliferation of pulmonary arterioles, induces vasculogenesis [22,23].

Studies have suggested that IL-13, an important mediator of granulomatous and vascular responses in the schistosomotic infection, acts in conjunction with the TGF-beta system, and its increased activity has been found in animals with Sch-PAH [24-26]. Moreover, TGF-beta signaling is higher in mice exposed to *Schistosoma* and patients who have died of Sch-PAH than in control samples. Besides this, the increase in TGF-beta after *Schistosoma* exposure is dependent on IL-4 and IL-13 in the animal model [27].

The aim of this study was to assess the serum concentrations of TGF-beta1 and IL-13 in patients with Sch-PAH and compare them with patients with schistosomiasis without PAH.

## Methods

### Study population, participants, ethical approval and exclusion criteria

This was a descriptive study with comparative groups. Patients with schistosomal periportal fibrosis and PAH confirmed by RHC (mean PAP ≥ 25 mmHg and pulmonary capillary wedge pressure ≤ 15 mmHg) were recruited from the pulmonary hypertension referral center at the Pronto Socorro Cardiológico de Pernambuco, Recife, Brazil. The comparative group consisted of patients with schistosomal periportal fibrosis without pulmonary hypertension assessed by Transthoracic Doppler echocardiography and matched by sex and age, from the schistosomiasis outpatient clinic at the Hospital das Clínicas, Universidade Federal de Pernambuco, Recife, Brazil.

Patients were selected from July 2010 to December 2012, and the study was approved by the Research Ethics Committee of the Universidade Federal de Pernambuco (registration number at SISNEP Fr-326154/CAAE-0096.0.172.000-10/REGISTRATION CEP/CCS/UFPE 099/10). All patients received an explanation concerning the scope of the study, and signed an Informed Consent Form. This study was conducted according to the Helsinki Declaration.

Exclusion criteria were: age ≤ 18 years of age; a history of alcohol abuse (alcohol intake in excess of 210 g/week for males and 140 g/week for females over the previous 5 years) [28]; evidence of non-schistosomal liver disease by ultrasound or clinical examination; hepatitis B or C viruses and/or HIV infection; evidence of congenital heart diseases (i.e. atrial septal defects); left-sided heart disease; moderate or severe lung disease (forced vital capacity [FVC] and/or forced expired volume in one second [FEV1] and/or FEV1/VFC ≤ 60% of predicted); and/or diagnosis of connective tissue disease.

### Diagnosis of PAH and schistosomiasis

All patients were previously submitted to abdominal ultrasound in order to define the presence of schistosomal periportal fibrosis, which was classified according to Niamey criteria [29] as: C-peripheral, D-central, E-advanced and F-very advanced (Siemens Acuson X150® with3,5 mHz convex transducers). The groups with and without PAH were compared in relation to each pattern of fibrosis.

The included patients with periportal fibrosis presented with the hepatointestinal (spleen longitudinal diameter < 120 mm) and hepatosplenic forms of schistosomiasis (spleen longitudinal diameter of ≥120 mm). The diameters of the porta and splenic veins were considered enlarged if they exceeded 12 mm and 9 mm, respectively. The presence of collateral vessels was also reported.

A Doppler echocardiography was performed on the comparative group to exclude PAH, using standard procedures [30] (Philips EnVisor C® system with 2.5 and 3.5 mHz mHz transducers). The following parameters were considered in order to exclude PAH: tricuspid regurgitation velocity ≤ 2.8 m/s, pulmonary artery systolic pressure ≤ 36 mmHg and no additional echocardiographic variables suggestive of pulmonary hypertension (increased velocity of pulmonary valve regurgitation, short acceleration time of right ventricular ejection into pulmonary artery, increased dimensions of right heart chambers, abnormal shapes and function of the interventricular septum, increased right ventricular wall thickness, and dilated pulmonary artery) [1].

The functional classification of patients with PAH was determined according to the modified World Health Organization classification from the New York Heart Association [31]. Those patients with PAH were previously submitted to RHC. Access to the venous and femoral arteries was obtained using the Seldinger technique. Catheterization was performed in the right and left chambers of the aorta, and manometry was conducted on the left ventricle, pulmonary artery, right ventricle and right atrium using a 06-French pigtail catheter and a 07-French wedge catheter for the occluded capillary wedge pressure. The output and resistance calculations were performed using the Fick method [32].

### Sample collection and assay

Venous blood samples were collected under aseptic conditions, using vacuum tubes (BD, Becton Dickinson, UK) without anticoagulants. Samples were centrifuged for 5 min at 2000 × g and the serum was stored in 0.5 ml aliquots at -80°C until assayed. The levels of TGF-beta1 and IL-13 were assessed using a sandwich enzyme-linked immunosorbent assay (ELISA) (R&D Systems, Inc, Minneapolis, MN). To activate latent TGF-beta1 to the immunoreactive form, 1 N HCl was used for acid activation and 1.2 N NaOH/0.5 M HEPES for neutralization. After activation, the samples (serum) were diluted and the concentration was measured according to the ELISA kit. The presence of cytokines was revealed and the results were expressed as pg/ml in accordance to standard curves. The assay sensitivity to TGF-beta1 was 15.4 pg/ml and to IL-13 57.4 pg/ml.

### Statistical analysis

The data was incorporated into the database Epi Info 3.02 and analysed using STATA. Results were presented as median and percentiles (interquartile range). Comparisons for quantitative variables were conducted using the Student t-test for independent samples, the Mann–Whitney test for nonparametric variables, and the Pearson correlation test for correlation analyses. The Fisher Chi-squared test was used to compare the categorical variables: sex, clinical form of schistosomiasis and periportal fibrosis pattern between the patients with and without PAH. A *p* value <0.05 was considered as statistically significant.

## Results

Thirty four patients with Sch-PAH and periportal fibrosis and 34 patients with periportal fibrosis without PAH diagnosed by Doppler echocardiography and matched by sex and age, concluded the study. One female with FEV1/VFC ≤ 60% and one male who was HbsAg antigen positive were excluded from the control group. Two women with PAH were also excluded: one presented with a previous diagnosis of cutaneous scleroderma and the other with atrial septal defect. The clinical and hemodynamic variables of the patients with Sch- PAH are shown in Table 1. Twenty seven (79.4%) patients were taking medication for PAH at the time of inclusion: 25 were on phosphodiesterase inhibitors, one on bosentan and one on calcium channel blockers (Table 1). There was no difference between groups regarding age, sex, the periportal fibrosis pattern and portal vein diameter (Table 2). The longitudinal diameter of the spleen was wider in the control group, without PAH. All patients in the control group and 30 patients with Sch-PAH presented the hepatosplenic form of schistosomiasis and only four patients in the Sch-PAH group presented the hepatointestinal form of the disease (Table 2).

**Table 1:** Clinical and hemodynamic characteristics of patients with schistosomiasis-associated pulmonary arterial hypertension

**Variables**	**Patients**	**Characteristics**
Functional class I/II/III/IV/N	34	9/10/7/8
Drugs to PAH/N (%)	34	27 (79.4%)
Mean PAP mmHg median (P25; P75)	34	55.5 (46; 62.7)
RAP mmHg median (P_25_; P_75_)	29	13 (9; 16)
LVEDP mmHg median (P_25_; P_75_)	32	14 (11.7; 15)
CI l/min/m^2^ median (P_25_; P_75_)	25	2.68 (1.97; 3.67)
PVR Dyn/sec/cm median (P_25_; P_75_)	30	989.5 (644.5; 1417.5)

Recife, 2012.

CI – cardiac index; LVEDP-left ventricular end-diastolic blood pressure; PAP- pulmonary artery pressure; N - sample size; PAH – pulmonary arterial hypertension; PVR- pulmonary vascular resistance; RAP- right arterial pressure.

**Table 2:** Comparison of patient characteristics according to the condition of schistosomiasis-associated pulmonary arterial hypertension

**Groups**	**General**	**With Sch-PAH**	**Without Sch-PAH**	**p-value**
Samples	68	34	34	-
Age (mean ± sd)	49.3 ± 12.9	49.8 ± 13.2	49.8 ± 12.8	0.759^a^
Sex: Male	24 (35.3%)	11 (32.3%)	13 (38.2%)	0.612
Clinical form				
Hepatosplenic	64 (94.1%)	30 (88.2%)	34 (100%)	0.039^c^
Hepatointestinal	04 (5.9%)	04 (17.8%)	00 (-)	
Periportal fibrosis				
D^x^	24 (35.3%)	13 (38.2%)	11 (32.3%)	
E	37 (54.4%)	18 (52.9%)	19 (55.9%)	0.674^c^
F	07 (10.3%)	03 (8.9%)	04 (11.8%)	0.685
TGF-β1 pg/ml (median – P_25_; P_75_)	19442.2 (11070.4; 30097.2)	22496.9 (15936.7; 32087.8)	13629.9 (10192.2; 22193.8)	0.006^b^
IL-13 pg/ml (median – P_25_; P_75_)	152.3 (100.2; 165.0)	152.3 (103.7; 177.8)	131.8 (100.2; 162.5)	0.177^b^
Portal vein diameter cm	1.15 ± 0.33	1.09 ± 0.37	1.23 ± 0.25	0.085^a^
Spleen diameter cm	14.5 ± 3.3	12.7 ± 2.17	16.4 ± 3.3	0.000^a^

Recife, 2012.

^a^Student’s t-test; ^b^Mann–Whitney nonparametric test; ^c^ Fisher X^2^ test; ^x^Reference value, D- central fibrosis; E- advanced fibrosis; F- very advanced fibrosis; TGF-β1 – transforming growth factor-beta 1; IL-13- interleukin-13.

Median serum levels of immunoreactive TGF-1 beta were significantly higher in patients with Sch-PAH compared to patients without PAH (22496.9 [IR: 15936.7 – 32087.8] pg/ml versus 13629.9 [IR: 10192.2 – 22193.8] pg/ml) (p = 0.006). There was no difference in the median value of IL-13 in the group with Sch-PAH (152.3 [IR: 103.7 - 177.8]) pg/ml, compared to patients without PAH (131.8 [IR: 100.2 - 162.5]) pg/ml (p = 0.177) (Table 2).

## Discussion

The present study has demonstrated significantly higher serum levels of immunoreactive TGF-beta1 in patients with Sch-PAH compared with patients with schistosomiasis without PAH. TGF-beta 1 is one isoform of TGF-beta that causes enhanced cell proliferation in smooth muscle cells in the pulmonary arteries of patients with idiopathic PAH and has a growth-inhibitory effect on normal cells [33]. A previous study has already demonstrated elevated levels of TGF-beta1 and other growth factors in serum samples of 46 patients with PAH with other diverse etiologies compared to 20 controls, and suggested that these substances may contribute to vascular remodeling in PAH [34].

The link between TGF-beta and the pathogenesis of Sch-PAH has been studied in animal models. The altered signaling of the TGF-beta system was demonstrated in experimental mouse models infected with *Schistosoma* and in lung specimens obtained from autopsies of individuals who had died of Sch-PAH through increased Smad2/3 activity found in the affected vessels [19]. Smads are proteins that can be phosphorylated after the attachment of TGF-beta in cell receptors, and which are able to move to the nucleus of the cells, altering essential functions [22]. The TGF-beta system is a probable pathway to direct the proliferation of pulmonary arterioles in PAH, leading to vasculogenesis, as well as intimal hyperplasia and growth of the media layer [21-23]. It is believed that in the case of PAH linked with BMPR-II mutation, the main mechanism is the imbalance secondary to the loss of BMPR-II function, associated with the increased activation of the TGF-beta superfamily of receptors [22].

Studies using the inhibition of TGF-beta in experimental models of PAH have confirmed the role of this growth factor in the pathogenesis of PAH and have indicated its possible use in the treatment of this disease [35-37]. PAH was induced in rats by monocrotaline injection, and the later treatment with an antibody against TGF-beta-ligand decreased the pulmonary artery systolic pressure and right ventricular hypertrophy, increased exercise capacity and reduced pulmonary vascular remodeling as evidenced by decreased vessel-wall thickness and number of pre-capillary arterioles. This effect occurred with bosentan, the endothelin receptor blocker. TGF-beta regulates endothelin-1 synthesis, which is a possible mediator of TGF-beta actions [38]. More recently, Graham et al. [27] demonstrated that a mouse model of Sch-PAH submitted to pharmacological blockage of the TGF-beta ligand and receptor and that mice lacking Smad3 were significantly protected from pulmonary vascular remodeling and PAH. This blockage also led to a decrease in IL-4 and IL-13 concentrations [27]. A significant increase was encountered in the levels of TGF-beta1 mRNA as measured by RNA sequencing in *S. mansoni*-exposed mice compared with unexposed mice, and there was no change in the TGF-beta2 and TGF-beta3 expressions with *S. mansoni* exposure. A more extensive expression was observed of phosphor-Smad2/3 in the pulmonary vascular intimal, medial, and adventitial regions in experimental Sch-PH lungs compared with control mice. These authors also demonstrated an increased expression of Smad2/3 within the pulmonary vascular media compartment in subjects who had died of Sch-PAH compared with control human lungs [27]. Accordingly, the present study also measured the TGF-beta1 isoform, and our results were similar, in that they supported a role played by this growth factor in the pathogenesis of Sch-PAH.

There was no difference in the median value of IL-13 in the group with Sch-PAH, compared to patients without PAH, contrary to that observed in animal models. Crosby et al. [26], using mice experimentally infected with Schistosoma, discovered that the grade of pulmonary vascular remodeling correlated with the egg burden in the lungs and with plasma Th1 and Th2 cytokines. Furthermore, IL-13 stimulated the migration of mouse pulmonary artery smooth muscle cells in transwell assays. There was a peak of cytokines IL-10, IL-13, IL-6 and IL-4, 17 weeks after infection [26]. In this animal model, transcutaneous infection of the animals with cercariae before the intravenousinjection of *Schistosoma* eggs some weeks later was required for PAH development, suggesting that a potent inflammatory response in the lungs due to prior sensitization is necessary to elicit PAH, more than the mere embolization of eggs in the lungs [25,26].

IL-13 signaling is mediated by a complex receptor system [39]. Some studies suggest that it is the balance between levels of IL-13Rα1 versus IL-13Rα2 that regulates the IL-13 mediated response. Graham et al. [25] demonstrated that vascular remodeling was reduced in mice with the loss of IL-13Rα1 receptors, which leads to the loss of IL-13 function, in a non-significant manner. On the other hand, mice without IL-13Rα2, and with an IL-13 gain-of-function, presented thicker intimal layers in the pulmonary vessels as well as higher right ventricular pressure. The increased IL-13 signaling through the loss of IL-13Rα2 receptors was able to elicit PAH in this model. Other authors have already reported that the imbalance in IL-13 receptors is the key feature for changing IL-13 signaling in other animal models of PAH and in patients with idiopathic PAH, more than changes in circulating levels of this cytokine [40,41]. Maybe, for this reason, the present study failed to demonstrate increased serum levels of IL-13 in patients with Sch-PAH. More recently, it was demonstrated that increased levels of IL-4 and IL-13 mRNA and proteins seen after exposure to *S. mansoni*, tended toward suppression after blockage of the TGF-β signaling pathway. These data suggest that IL-4/ IL-13 and TGF-β act mutually [27].

Although parasite eggs and *S. mansoni-*soluble egg antigens were found in the lungs of experimentally infected mice and in specimens of human intestine, there was no significant amount of parasite-derived antigens within the lungs of individuals who had died of Sch-PAH, despite the presence of pulmonary vascular remodeling with plexiform lesions and arterial medial thickening [19]. This suggests that after an initial acute inflammatory process, which may be triggered when the schistosomula passes through the lungs in genetically predisposed individuals, vascular remodeling is established and can progress or persist regardless of the presence of the antigen. Maybe this process starts and progresses with the release of cytokines and growth factors by the granulomas produced in other sites, such as the liver.

Crosby et al. [42] demonstrated that treatment with praziquantel was effective in eradicating adult worms of *S. mansoni*, prevented PAH development and reversed pulmonary vascular remodeling in infected mice. Contrary to what occurs with liver fibrosis, which may reverse or reduce with parasite treatment; this does not seem to occur with the lung pathology of humans [19,43,44]. For all these reasons it is important to clarify the immune mechanisms involved in the pathogenesis of Sch-PAH, in order to discover more effective therapy targets, acting directly on the proliferative component of PAH.

### Study limitations

The sample size in the present study was probably small, but if we consider that this study enrolled individuals with PAH of one single etiology, it was an important sample of patients. TGF-beta1 and IL-13 were chosen according to the new evidence encountered in studies with animal models of Sch-PAH. This was a preliminary study, therefore new studies using larger samples and evaluating other growth factors and cytokines need to be performed to confirm our results and bring new insights into the immunopathogenesis of this devastating disease.

## Conclusions

This study has demonstrated significantly increased serum levels of TGF-beta1 in patients with Sch-PAH compared with patients with schistosomiasis without PAH, suggesting that this growth factor may contribute to vascular remodeling in this disease. However, this study was not able to detect a significantly elevated level of IL-13 in patients with Sch-PAH, maybe because either a larger sample size is necessary or an alteration in the IL-13 receptors is responsible for the augmented IL-13 signaling found in animal models.

## Abbreviations

BMPR-II: Bone morphogenetic protein receptor type II; FEV1: Forced expired volume in one second; FVC: Forced vital capacity; IL: Interleukin; PAH: Pulmonary arterial hypertension; PAP: Pulmonary artery pressure; RHC: Right heart catheterization; Sch-PAH: Schistosomiasis-associated pulmonary arterial hypertension; TGF-beta: Transforming growth factor beta; Th: T helper.

## Competing interest

The authors reported the following competing interest: Dr. Bandeira received a speech fee from Pfizer.

## Authors’ contributions

RCSF, SMLM, ALCD, APB contributed to the conception and design of this study, the acquisition of patients data, analysis and interpretation of data and were involved in drafting the manuscript. SMLM carried out the imunnoassays. The other authors (CAMS, LACL, CAP, IMF, ABM, MOA) contributed to the acquisition of data and clinical specimens. All authors have given approval for the final version of the manuscript to be published.

## Pre-publication history

The pre-publication history for this paper can be accessed here:

http://www.biomedcentral.com/1471-2334/14/282/prepub

## References

[B1] Galiè N Hoeper MM Humbert M Torbicki A Vachiery J Barbera JA Beghetti M Corris P Gaine S Gibbs JS Gomez-Sanchez MA Jondeau G Klepetko W Opitz C Peacock A Rubin L Zellweger M Simonneau G Guidelines for diagnosis and treatment of pulmonary hypertension. The Task Force for the diagnosis and treatment of pulmonary hypertension of the European Society of Cardiology (ESC) and the European Respiratory Society (ERS) endorsed by the International Society of Heart and Lung Transplantation (ISHLT) Eur Respir J 2009 14 1219 126319749199

[B2] Simonneau G Gatzoulis MA Adatia I Celermajer D Denton C Ghofrani A Sanchez MAG Kumar RK Landzberg M Machado RF Olschewski H Robbins IM Souza R Updated clinical classification of pulmonary hypertension J Am Coll Cardiol 2013 14 25 Suppl D34 D41 24355639 10.1016/j.jacc.2013.10.029

[B3] Graham BB Bandeira AP Morrell NW Butrous G Tuder RM Schistosomiasis-associated pulmonary hypertension: pulmonary vascular disease: the global perspective Chest 2010 14 20S 29S 10.1378/chest.10-0048 20522577 PMC5989787

[B4] Chitsulo L Engels D Montresor A Savioloi L The global status of schistosomiasis and its control Acta Trop 2000 14 1 41 51 10.1016/S0001-706X(00)00122-4 10996119 PMC5633072

[B5] Ross AG Bartley PB Sleigh AC Olds GR Li Y Williams GM McManus DP Schistosomiasis N Engl J Med 2002 14 1212 1220 10.1056/NEJMra012396 11961151

[B6] Ward TJC Fenwick A Butrous G The prevalence of pulmonary hypertension in schistosomiais: a systematic review PVRI Rev 2011 14 1 12 21 10.4103/0974-6013.85614

[B7] Ferreira RC Domingues AL Bandeira AP Markman-Filho B Albuquerque-Filho ES de Correia Araújo AC Batista LJB Markman M Campelo ARL Prevalence of pulmonary hypertension in patients with schistosomal liver fibrosis Ann Trop Med Parasitol 2009 14 129 143 10.1179/136485909X398168 19208297

[B8] Lapa M Dias B Jardim C Fernandes CJ Dourado PM Figueiredo M Farias A Tsutsui J Terra-Filho M Humbert M Souza R Cardiopulmonary manifestations of hepatosplenic schistosomiasis Circulation 2009 14 1518 1523 10.1161/CIRCULATIONAHA.108.803221 19273723

[B9] Gryseels B Polman K Clerinx J Kestens L Human schistosomiasis Lancet 2006 14 1106 1118 10.1016/S0140-6736(06)69440-3 16997665

[B10] Abath FGC Morais CNL Montenegro CEL Wynn TH Montenegro SML Immunopathogenic mechanisms in schistosomiasis: what can be learnt from human studies? Trends Parasitol 2006 14 2 85 91 10.1016/j.pt.2005.12.004 16380294

[B11] Morais CNL Souza JR Melo WG Aroucha ML Miranda P Domingues ALC Abath FGC Montenegro SML Cytokine profile associated with chronic and acute human schistosomiasis mansoni Mem Inst Oswaldo Cruz 2008 14 6 561 568 10.1590/S0074-02762008000600009 18949326

[B12] Wilson MS Mentink-Kane MM Pesce JT Ramalingam TR Thompson R Wynn TA Immunopathology of schistosomiasis Immunol Cell Biol 2007 14 2 148 154 10.1038/sj.icb.7100014 17160074 PMC3437548

[B13] Caldas RC Campi-Azevedo AC Oliveira LFA Silveira AMS Oliveira RC Gazzinelli G Human schistosomiasis mansoni: immune responses during acute and chronic phases of the infection Acta Trop 2008 14 2–3 109 117 18577364 10.1016/j.actatropica.2008.05.027

[B14] Yu L Sun X Yang F Yang J Shen J Wu Z Inflammatory cytokines IFN-Ƴ, IL-4, IL-13 and TNF-α alterations in schistosomiasis: a meta-analysis Parasitol Res 2012 14 4 1547 1552 10.1007/s00436-011-2661-4 21968955

[B15] Warren KS Hepatosplenic schistosomiasis: a great neglected disease of the liver Gut 1978 14 572 577 10.1136/gut.19.6.572 355069 PMC1412035

[B16] Domingues ALC Ferraz AAB Coelho J Doença Hepática Esquistossomótica Aparelho Digestivo: Clínica e Cirurgia 2012 São Paulo: Editora Atheneu 1559 1575

[B17] Strauss E Hepatosplenic schistosomiasis: a model for the study of portal hypertension Ann Hepatol 2002 14 1 6 11 15114290

[B18] Morris W Knauer CM Cardiopulmonary manifestations of schistosomiasis Semin Respir Infect 1997 14 2 159 170 9195681

[B19] Graham BB Chabon J Bandeira A Espinheira L Butrous G Tuder RM Significant intrapulmonary schistosoma egg antigens are not present in schistosomiasis-associated pulmonary hypertension Pulm Circ 2011 14 4 456 461 10.4103/2045-8932.93544 22530100 PMC3329075

[B20] Tuder RM Pathology of pulmonary arterial hypertension Semin Respir Crit Care Med 2009 14 376 385 10.1055/s-0029-1233307 19634077

[B21] Nicod LP The endothelium and genetics in pulmonary arterial hypertension Swiiss Med Wkly 2007 14 437 442 10.4414/smw.2007.11668 17705107

[B22] Newman JH Phillips JA 3rd Loyd JE Narrative review: the enigma of pulmonary arterial hypertension: new insights from genetic studies Ann Intern Med 2008 14 278 283 10.7326/0003-4819-148-4-200802190-00006 18283205

[B23] Goumans MJ Liu Z ten Dijke P TGF-β signaling in vascular biology and disfunction Cell Res 2009 14 116 127 10.1038/cr.2008.326 19114994

[B24] Price LC Wort SJ Perros F Dormüller P Huertas A Montani D Cohen-Kaminsky S Humbert M Inflammation in pulmonary arterial hypertension Chest 2012 14 1 210 221 10.1378/chest.11-0793 22215829

[B25] Graham BB Mentink-Kane MM El-Haddad H Purnell S Zhang L Zaiman A Redente EF Riches DWH Hassoun PM Bandeira AG Champion HC Butrous G Wynn TA Tuder RM Schistosomiaisis-induced experimental pulmonary hypertension. role of interleukin-13 signaling Amer J Pathol 2010 14 3 1549 1561 10.2353/ajpath.2010.100063 20671265 PMC2928984

[B26] Crosby A Jones FM Southwood M Stewart S Schermuly R Butrous G Dunne DW Morrel NW Pulmonary vascular remodeling correlates with lung eggs and cytokines in murine schistosomiasis Am J Respir Crit Care Med 2010 14 279 288 10.1164/rccm.200903-0355OC 19965814

[B27] Graham BB Chabon J Gebreab L Poole J Debella E Davis L Tanaka T Sanders L Dropcho N Bandeira A Vandivier RW Champion HC Butrous G Wang X Wynn TA Tuder RM Transforming growth factor-β signaling promotes pulmonary hypertension caused by *Schistosoma mansoni* Circulation 2013 14 1354 1364 10.1161/CIRCULATIONAHA.113.003072 23958565 PMC3880024

[B28] Walsh K Alexander G Alcoholic liver disease Postgrad Med J 2000 14 280 286 10.1136/pmj.76.895.280 10775280 PMC1741594

[B29] Richter J Domingues ALC Barata CH Prata AR Lambertucci JR Report of the second satellite symposium on ultrasound in schistosomiasis Mem Inst Oswaldo Cruz 2001 14 Suppl 151 156 11586442 10.1590/s0074-02762001000900023

[B30] Lang RM Bierig M Devereux RB Flachskampf FA Foster E Pellikka PA Picard MH Roman MJ Seward J Shanewise JS Solomon SD Spencer KT Sutton MS Stewart WJ Recommendations for chamber quantification: a report from the American Society of Echocardiography’s Guidelines and Standards Committee and the Chamber Quantification Writing Group, Developed in Conjunction with the European Association of Echocardiography, a Branch of the European Society of Cardiology J Am Soc Echocardiogr 2005 14 12 1440 1463 10.1016/j.echo.2005.10.005 16376782

[B31] Barst RJ McGoon M Torbicki A Sitbon O Krowka MJ Olschewski H Gaine S Diagnosis and differential assessment of pulmonary arterial hypertension J Am Coll Cardiol 2004 14 12 40S 47S 10.1016/j.jacc.2004.02.032 15194177

[B32] Gupta H Ghimire G Naeije R The value of tools to assess pulmonary arterial hypertension Eur Respir Rev 2011 14 122 222 235 10.1183/09059180.00006911 22130815 PMC9487750

[B33] Morrel NW Yang X Upton PD Jourdan KB Morgan N Sheares KK Trembath RC Altered growth responses of pulmonary artery smooth muscle cells from patients with primary pulmonary hypertension to transforming growth factor-β1 and bone morphogenetic protein Circulation 2001 14 790 795 10.1161/hc3201.094152 11502704

[B34] Selimovic N Bergh CH Anderson B Sakiniene E Carlsten H Rundqvist B Growth factors and interleukin-6 across the lung circulation in pulmonary hypertension Eur Respir J 2009 14 3 662 668 10.1183/09031936.00174908 19324949

[B35] Zaiman AL Podowski M Medicherla S Gordy K Xu F Zhen L Shimoda LA Neptune E Higgins L Murphy A Chakravarty S Protter A Sehgal PB Champion HC Tuder RM Role of TGF-β-beta/Alk5 signaling pathway in monocrotaline-induced pulmonary hypertension Am J Respir Crit Care Med 2008 14 896 905 10.1164/rccm.200707-1083OC 18202349 PMC2292828

[B36] Long L Crosby A Yang X Southwood M Upton PD Kim DK Morrell NW Altered bone morphogenetic protein and transforming growth factor-beta signaling in rat models of pulmonary hypertension: potential for activin receptor-like kinase-5 inhibition in prevention and progression of disease Circulation 2009 14 566 576 10.1161/CIRCULATIONAHA.108.821504 19153267

[B37] Megalou AJ Glava C Oikonomidis DL Vilatei A Agelaki MG Baltogiannis GG Papalois A Vlahos AP Kolettis TM Transforming growth factor-beta inhibition attenuates pulmonary arterial hypertension in rats Int J Clin Exp Med 2011 14 332 340 PMC2971541 21072267

[B38] Star GP Giovinazzo M Langleben D Effects of bone morphogenic proteins and tranforming growth factor-beta on in-vitro production of endothelin-1 by human pulmonary microvascular endothelial cells Vascul Pharmacol 2009 14 1-2 45 50 10.1016/j.vph.2008.09.001 18849010

[B39] Mentink-Kane MM Wynn TA Opposing roles for IL-13 and IL-13 receptor alpha 2 in health and disease Immunol Rev 2004 14 191 202 10.1111/j.0105-2896.2004.00210.x 15546394

[B40] Daley E Emson C Guignabert C de Wall MR Louten J Kurup VP Hogaboam C Taraseviciene-Stewart L Voelkel NF Rabinovitch M Grunig E Grunig G Pulmonary arterial remodeling induced by a Th2 immune response J Exp Med 2008 14 361 372 10.1084/jem.20071008 18227220 PMC2271018

[B41] Hecker M Zastona Z Kwapiszewska G Niess G Zakrzewicz A Hergenreider E Wilhelm J Marsh LM Sedding D Klepetko W Lohmeyer J Dimmeler S Seeger W Weismann N Schermuly RT Kneidinger N Eickelberg O Morty RE Dysregulation of the IL-13 receptor system. a novel pathomechanism in pulmonary arterial hypertension Am J Resp Crit Care Med 2010 14 805 818 10.1164/rccm.200909-1367OC 20522789

[B42] Crosby A Jones FM Kolosionek E Southwood M Purvis I Soon E Butrous G Dunne DE Morrell NW Praziquantel reverses pulmonary hypertension and vascular remodeling in murine schistosomiasis Am J Resp Crit Care Med 2011 14 4 467 473 10.1164/rccm.201101-0146OC 21659614

[B43] Andrade ZA Regression of hepatic fibrosis Rev Soc Bras Med Trop 2005 14 6 514 520 10.1590/S0037-86822005000600013 16410929

[B44] Ruiz-Guevara R Noya BA Valero SK Lecuna P Garassini M Noya O Clinical and ultrasound findings before and after praziquantel treatment among Venezuelan schistosomotic patients Rev Soc Bras Med Trop 2007 14 5 505 511 10.1590/S0037-86822007000500003 17992403

